# Editorial

**Published:** 2013-04-26

**Authors:** Nikhil Marwah

## Abstract

In the dynamics of this world ever since centuries, one thing has always remained constant and that is ‘change’. The history of entire human race is a living example of this evolution. We, in the publication world, are no different and hence the dynamics always go on.

## A New Beginning...

In the dynamics of this world ever since centuries, one thing has always remained constant and that is ‘change’. The history of entire human race is a living example of this evolution. We, in the publication world, are no different and hence the dynamics always go on. As a part of this humble beginning of this new editorial board and editors, I on behalf of the entire board express my whole hearted gratitude to erstwhile editors, especially the journal's founding editor Dr Usha Mohan Das, who has done a stupendous job to initiate the journal and get it to its present status. We will carry the baton forward and hope to accomplish all our objective of academic excellence for the journal in the time to come. The new board will carry on the academic excellence initiated by this journal and also improve upon the shortcomings that were encountered in the past. As a part of our renewed approach, we would be taking out one special issue of IJCPD each year which would focus on specific areas of pediatric dentistry. We assure you a promising approach to the literature of dentistry illuminating the world of knowledge for our contributors and subscribers thus committed to produce the higher standards.

Let's begin with a new motto... Amelioration of Knowledge...


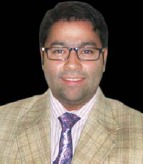
**Nikhil Marwah**
Editor-in-chief


